# A multidimensional examination of academic procrastination reasons in pre-service teachers: SEM and latent profile analysis on the structural role of self-confidence-based procrastination

**DOI:** 10.3389/fpsyg.2026.1856110

**Published:** 2026-08-27

**Authors:** Mine Çeliköz

**Affiliations:** Department of Educational Sciences, Yıldız Technical University, İstanbul, Türkiye

**Keywords:** academic procrastination, academic self-confidence, active procrastination, latent profile analysis, pre-service teachers, procrastination reasons, structural equation modeling

## Abstract

Academic procrastination in teacher education carries implications extending beyond individual performance to future pedagogical practice and student outcomes. This study examined procrastination dynamics among preservice teachers through an integrated framework encompassing the predictive role of personal variables, the structural position of confidence-based procrastination, and latent profile heterogeneity. This cross-sectional study was conducted with 490 preservice teachers enrolled in seven primary education programs. Data were collected via a six-dimensional Academic Procrastination Reasons Scale and personal variables form. Structural equation modeling and latent profile analysis were performed using RStudio. Procrastination reasons exhibited moderate levels and positive intercorrelations across subscales. Personal variables were selectively associated with procrastination dimensions. No personal variable was significantly associated with confidence-based procrastination; however, academic self-confidence-based reasons were strongly and independently associated with all procrastination dimensions. Latent profile analysis identified four distinct profiles: *Selective Procrastinators* (15%), *Moderate Procrastinators* (44%), *High-Engaged Procrastinators* (20%), and *Minimal Procrastinators* (22%). Integrated findings demonstrated that procrastination based on academic self-confidence may hold a structurally privileged position over all other procrastination reasons, independent of demographic predictors. This finding, which places academic self-confidence at the center of academic procrastination, provides a unique theoretical contribution to research on motivation, self-beliefs, and academic behavior. The consistent emergence of this pattern in both variable-centered and person-centered analyses suggests that procrastination interventions in teacher education should be profile-based and academic confidence-focused, instead of relying on demographic grouping. Accordingly, specific recommendations are presented within the scope of the study.

## 1 Introduction

As procrastination evolves into a chronic behavioral pattern through repetition, students inevitably face a multi-layered cycle of detriment. Numerous studies demonstrate that habitual procrastination leads to a decline in academic achievement and motivation (Hidalgo-Fuentes et al., 2024; Imhof et al., 2021; Kim and Seo, 2015; Steel, 2007), while increasing academic misconduct (Ghayas et al., 2022; Siaputra, 2013) and serious issues extending to school dropout (Husain et al., 2025; Rad et al., 2023). In the long term, the effects of procrastination manifest as cumulative problems that trigger one another over time. Ultimately, this process systematically undermines an individual’s career development, professional competence, and overall life satisfaction (Gueorguieva, 2011; Liang and Bautista, 2025), evolving into a persistent source of psychological burden and burnout across all life domains.

Although these negative patterns are predominantly associated with “passive procrastination” in the literature, not all forms of procrastination stem from the same psychological dynamics. In some cases, procrastination emerges as a deliberate and strategic delay, conceptualized as “active procrastination” (Chowdhury and Pychyl, 2018; Klingsieck, 2013). However, the conditions under which this distinction leads to functional or maladaptive outcomes remain unclear. While academic procrastination is significant for all students, it is particularly critical for pre-service teachers (Martín-Antón et al., 2024), with studies highlighting its prevalence in this population (Arazo et al., 2023; Casama et al., 2026). As this group is expected to serve as future role models in skills such as time management, self-regulation, and academic competence, understanding the structural and multidimensional reasons behind their procrastination, rather than just its level, is essential.

The literature on academic procrastination among pre-service teachers primarily focuses on a limited number of variables and variable-centered approaches. Consequently, these perspectives remain insufficient in explaining the multidimensional nature of procrastination and distinct inter-individual patterns. Specifically, how procrastination reasons, such as teaching approach, locus of control, course content, academic self-confidence, and academic inadequacy, interact, and the mediating role of confidence-based reasons, have not been sufficiently elucidated. Furthermore, person-centered studies identifying student profiles based on procrastination reasons remain remarkably scarce.

This study integrates variable-centered and person-centered approaches to investigate academic procrastination. Within this framework, (a) the reasons for academic procrastination are examined across six dimensions; (b) the direct and indirect effects of personal variables on these dimensions, along with the mediating role of academic self-confidence, are tested using structural equation modeling (SEM); and (c) distinct procrastination patterns are identified through latent profile analysis (LPA). This holistic approach is expected to elucidate the multi-layered nature of academic procrastination, providing theoretical and practical contributions to targeted interventions in the context of teacher education.

## 2 Literature review

### 2.1 Theoretical framework of academic procrastination

Addressing academic procrastination through superficial explanations such as poor time management or laziness overlooks its multidimensional nature. Procrastination is a complex process shaped by the interaction of students’ task value, self-efficacy beliefs, emotional responses, and contextual conditions (Solomon and Rothblum, 1984; Herut and Gorfu, 2024; Ramadhani et al., 2026; Svartdal et al., 2020). Therefore, procrastination tendencies should be evaluated as dynamic patterns emerging at the intersection of individual characteristics and the social environment.

The primary theoretical approaches explaining this process in the literature are complementary. *Temporal Motivation Theory* proposes that procrastination intensifies as the delay of reward increases and the allure of immediate gratification grows (Steel and König, 2006). *Emotion Regulation Theory* treats procrastination as a coping strategy to avoid negative emotions such as anxiety, boredom, and inadequacy (Sirois and Pychyl, 2013). Furthermore, *Self-Regulation Theory* emphasizes that deficiencies in planning, monitoring, and evaluation processes heighten procrastination (Fernie et al., 2016; Häfner et al., 2014), as individuals with weak self-regulation and time-management skills are more prone to academic procrastination (Steel, 2007; Wolters and Brady, 2021). In this context, time management serves as both a component of self-regulation processes and a critical mechanism directly influencing the formation of procrastination behavior.

### 2.2 Teaching approach (TA) and procrastination

Teaching approach refers to the fundamental pedagogical orientation regarding how a teacher structures instruction and directly shapes students’ learning experiences. Dissatisfaction with the instructional process can trigger task avoidance and procrastination tendencies. This relationship is best understood within the framework of *Self-Determination Theory (SDT),* which explains the core determinants of motivation (Codina et al., 2018; Ryan and Deci, 2000). According to *SDT*, satisfying the needs for autonomy, competence, and relatedness enhances intrinsic motivation and engagement in learning. In this context, teaching approaches are generally categorized as *autonomy-supportive* or *controlling* (Codina et al., 2020). Autonomy-supportive approaches strengthen motivation by providing choices, offering meaningful learning experiences, and considering individual differences, whereas controlling approaches undermine students’ perceived autonomy through pressure and coercive guidance (Reeve et al., 2004; Kaplan, 2018).

Autonomy-supportive approaches reduce procrastination by enhancing motivation and engagement; conversely, controlling approaches, characterized by pressure and excessive personal control, strengthen procrastination tendencies by undermining the sense of autonomy (Codina et al., 2018; Ryan and Deci, 2020). The literature indicates that autonomy-supportive teaching reduces procrastination, with variables such as self-efficacy, motivation, hope, and perceived structure mediating this relationship (Alexander and Onwuegbuzie, 2007; Bozgun and Baytemir, 2022; Mouratidis et al., 2024; Tisocco and Liporace, 2023). However, how this relationship is specifically shaped through internal mechanisms like academic self-confidence remains insufficiently elucidated. Therefore, examining teaching approaches alongside confidence-based procrastination provides a more comprehensive theoretical and practical framework for understanding procrastination behaviors among pre-service teachers.

### 2.3 Course content (CC) and procrastination

The course content factor reflects the perceived value of the course from the student’s perspective, their level of interest, and the extent to which they find the material personally relevant and appropriate. Students predominantly base their academic evaluations on the curriculum and learning activities; these perceptions directly influence their satisfaction and engagement levels. Perceiving course content as meaningful, useful, and personally relevant strengthens students’ intrinsic and extrinsic motivation, supports their beliefs in success, and increases active participation in the learning process. This condition is associated with a decrease in academic procrastination tendencies (Cheng and Xie, 2021; Kljajic et al., 2022; Lim et al., 2016). Conversely, a lack of interest in course content or the perception that it lacks personal utility weakens student satisfaction and engagement, which can increase procrastination behaviors (Bäulke and Dresel, 2023; Çelikoz and Seçer, 2018). Current literature indicates that course content is a critical variable shaping learning processes, perceptions of academic competence and confidence, and procrastination behaviors. Therefore, course content is treated as a fundamental determinant in explaining the multidimensional nature of academic procrastination.

### 2.4 Internal and external locus of control (IC-EC) and procrastination

*Locus of Control* is a fundamental cognitive orientation explaining how individuals attribute life outcomes (Rotter, 1966). Within this framework, individuals are positioned along a continuum between internal control, which attributes outcomes to personal effort, and external control, which assigns them to fate, luck, or external forces (Carden et al., 2004; Rotter, 1966). This orientation directly influences students’ engagement, motivation, and coping strategies.

Students with a high internal locus of control, believing they can influence their academic outcomes, tend to exert more effort, utilize self-regulation strategies, and actively participate in learning (Choy and Cheung, 2018; Grillo and Damacena, 2015; Nießen et al., 2022). Conversely, an external locus of control is associated with hopelessness, low motivation, and task avoidance, as failures are attributed to uncontrollable factors (Gangai et al., 2016). Accordingly, the literature consistently demonstrates that internal control is negatively, while external control is positively correlated with academic procrastination (Rakes et al., 2013; Wang et al., 2025). Furthermore, the impact of locus of control on academic success and learning outcomes is closely linked to individuals’ self-efficacy and self-evaluation processes (Bahl et al., 2025; Brownlow and Reasinger, 2000; Kader, 2022). In this context, it is anticipated that the influence of locus of control on pre-service teachers’ procrastination reasons may offer a more explanatory pattern when examined alongside confidence-based mechanisms.

### 2.5 Academic self-confidence (AC) and academic inadequacy (AI) in procrastination

Academic self-efficacy is recognized as one of the most powerful predictors of academic procrastination. According to Bandura (1997), individuals with low self-efficacy struggle to persist against challenges and easily lose their motivation. While these students delay starting academic tasks due to fear of failure (Steel, 2007; Wäschle et al., 2014), those confident in their academic performance may engage in intentional delay to deliver a successful performance at the last minute (Chu and Choi, 2005). This distinction marks a critical difference between passive (avoidance-based) and active (strategic) forms of procrastination (Shaked and Altarac, 2023). The literature generally indicates a negative correlation between academic self-efficacy and procrastination (Cutipa-Flores et al., 2025; Ferrari et al., 1992; Gregory et al., 2023). Low self-efficacy and the accompanying perception of academic inadequacy strengthen procrastination by triggering emotional processes such as anxiety, hopelessness, academic stress, and low self-esteem (Batool, 2020; Nie et al., 2025; Zhang et al., 2018). In this process, procrastination serves not only as a cognitive delay but also as a self-handicapping strategy to protect an individual’s self-worth (Awwad et al., 2022; Tice and Baumeister, 1997).

For active procrastinators who prioritize tasks and choose to delay to meet deadlines, academic self-confidence stands out as a core characteristic driving this behavior. Academic self-confidence is conceptualized as a stable and generalized belief in overall academic competence, showing a close conceptual overlap with the global self-evaluation dimension in Rosenberg’s (1965) self-esteem theory. Students with high academic self-confidence can perceive time pressure as a motivator rather than a threat, using task delay as a strategic choice (Chu and Choi, 2005; Meng and Liu, 2025). Research indicates that intentional procrastination is positively associated with adaptive outcomes such as academic hardiness, achievement, and well-being; while negatively correlated with anxiety and maladaptive coping (Bakar et al., 2022; Habelrih and Hicks, 2015; Kooren et al., 2024; Priya et al., 2025). However, the mechanisms through which personal variables shape these relationships, specifically the potential mediating role of academic self-confidence, remain empirically underexplored.

### 2.6 Academic procrastination profiles

Academic procrastination research is predominantly conducted through variable-centered approaches, such as correlation, regression, and structural equation modeling (*SEM*), which examine average relationships between variables. However, this approach may overlook inter-individual differences and heterogeneous subgroups within the sample (Howard and Hoffman, 2018; Spurk et al., 2020). Conversely, person-centered approaches aim to understand how academic behaviors manifest in different forms by identifying subgroups of individuals who share similar variable patterns (Bergman et al., 2003; Meyer et al., 2013). In this context, Latent Profile Analysis (*LPA*) provides a suitable method to reveal the multidimensional nature of academic procrastination and inter-individual differentiations (Grunschel et al., 2013; Zhou et al., 2024).

Existing studies have examined procrastination behavior under various profile structures. These profiles are categorized into levels varying by the intensity of procrastination (Rist et al., 2023; Rozental et al., 2015; Zhou et al., 2024), patterns based on emotional processes and self-regulation (Gross et al., 2024; Rebetez et al., 2015), and differentiations linked to motivational orientations and academic prioritization (Ozberk and Türk-Kurtça, 2021; Xu, 2022). Some studies define mixed profile structures that integrate these dimensions (Gross et al., 2024). However, these investigations have largely been conducted with samples other than pre-service teachers. Since the procrastination behaviors of pre-service teachers can affect not only their academic performance but also their future professional competence, identifying procrastination profiles specific to this group is of significant importance. This study aims to fill this gap by revealing profile structures based on the reasons for academic procrastination.

### 2.7 The present study

Integrating the theoretical and empirical literature, personal and contextual variables may shape academic procrastination not only through direct pathways but also via academic self-confidence perceptions. Accordingly, confidence-based procrastination is hypothesized to function as a mediating mechanism between personal variables and distinct procrastination reasons. Examining these relational patterns among pre-service teachers may further enable the identification of procrastination profiles specific to this population. Using both variable-centered (*SEM*) and person-centered (*LPA*) approaches, this study aims to comprehensively investigate the multidimensional structure of academic procrastination among pre-service teachers. The following hypotheses and research questions were tested accordingly.

*Hypotheses:*

*H1*: Personal variables (gender, grade level, and academic achievement) significantly predict dimensions of academic procrastination.

*H2*: Academic confidence-based reasons (AC) play a mediating role in the relationship between personal variables (gender, grade level, and academic achievement) and dimensions of academic procrastination.

*Research Questions:*

RQ1: How are the reasons for academic procrastination (TA, CC, IC, EC, AC, AI) distributed among pre-service teachers?

RQ2: How many different profiles can be identified based on the reasons for academic procrastination among pre-service teachers, and what characteristics do these profiles have?

## 3 Method

### 3.1 Research model

This study is a cross-sectional survey examining the reasons for academic procrastination among pre-service teachers across six dimensions. Variable-centered and person-centered approaches were used together to explain the relational structure of procrastination behaviors among variables and to reveal the differing academic procrastination profiles among pre-service teachers.

### 3.2 Participants and procedure

The participants in the study consisted of 490 pre-service teachers studying at various universities’ Faculties of Education in Turkey. Ethical approval was obtained prior to the commencement of the study. Data were collected via Google Forms, and the ethical principles of the Declaration of Helsinki were followed. Of the participants, 346 were female (70.6%) and 144 were male (29.4%). The distribution of participants by department is as follows: Mathematics Education (*n* = 178), Early Childhood Education (*n* = 58), Social Studies Education (*n* = 46), Computer and Instructional Technologies Education–BÖTE (*n* = 34), Classroom Teaching (*n* = 85), Language Teaching (*n* = 35), and Science Education (*n* = 54). The distribution by grade level is as follows: 1st grade (*n* = 122), 2nd grade (*n* = 157), 3rd grade (*n* = 118), and 4th grade (*n* = 93). Academic achievement levels were classified as poor (*n* = 32), average (*n* = 117), good (*n* = 229), and very good (*n* = 112). This distribution shows that the sample represents a variety of fields, grades, and achievement levels among pre-service teachers.

### 3.3 Data collection tool

The study was based on *the Academic Procrastination Reasons Scale* developed by Yeşil (2012), which originally consisted of 43 items. The reliability coefficients reported in the original study of the scale are high (*α* = 0.88), and the internal consistency values for the subscales are within acceptable ranges.

In the current study, preliminary analyses based on the original factor structure of the scale indicated that the fit of the measurement model could be improved in the current sample. This suggests that making the scale more sensitive and functional to different sample characteristics could enhance measurement sensitivity. In addition, the similarity of some items in terms of content and wording, the need to increase the readability and comprehensibility of the scale, and the desire to create a more parsimonious structure with items that represent each dimension most strongly from a theoretical perspective necessitated the development of a short form.

Accordingly, a scale refinement process based on exploratory and confirmatory factor analyses was conducted; items with high factor loadings and discriminative power were selected for each dimension, resulting in a six-dimensional, 18-item short form with three items per dimension. This short form was used in all subsequent analyses of the study.

The short form consists of six sub-dimensions: Teaching-approach-based reasons (TA), External-control-based reasons (EC), Internal-control-based reasons (IC), Course-content-based reasons (CC), Academic-confidence-based reasons (AC), and Academic-inadequacy-based reasons (AI). This structure aims to represent the cognitive, motivational, and self-regulatory mechanisms underlying academic procrastination in relation to learning processes in a multidimensional manner. All items on the scale end with the phrase “…causes me to procrastinate” and are answered using a 5-point Likert-type scale (1 = *Strongly disagree*, 5 = *Strongly agree*). Higher scores indicate that the relevant dimension contributes more strongly to procrastination behavior.

The Academic Confidence–based reasons (AC) dimension of the scale reflects a tendency toward procrastination (active procrastination) grounded in excessive confidence that the student will succeed despite leaving academic tasks to the last minute (e.g.*, “My success in the courses I study at the last-minute causes me to procrastinate”*). In contrast, the Academic-inadequacy-based reasons (AI) dimension expresses weak self-efficacy perceptions (passive procrastination) that the student’s early work will be ineffective or result in failure (e.g.*, “My fear that I will forget the information when I study early causes me to procrastinate”*).

### 3.4 Validity and reliability

The construct validity of the scale was tested using Confirmatory Factor Analysis (*CFA*). *CFA* results showed that the six-dimensional, 18-item measurement model fit the data well (*CFI* = 0.937, *RMSEA* = 0.043, *SRMR* = 0.047).

The internal consistency reliability coefficients of the scale are presented in Table 1.

**Table 1:** Reliability coefficients of the CFA-based short form.

Subscale	Number of items	Cronbach’s alpha *(α)*	McDonald’s omega *(ωₜ)*	Reliability level
Teaching approach	3	0.70	0.76	Good
External-control-based reasons	3	0.72	0.73	Acceptable
Internal-control-based reasons	3	0.75	0.74	Good
Course content	3	0.71	0.73	Good
Academic confidence	3	0.65	0.66	Acceptable
Academic inadequacy	3	0.66	0.67	Acceptable
General short form (18 items)	18	0.85	0.90	Very good

According to the findings, acceptable levels of internal consistency were achieved across all subscales of the scale. The highest reliability value was calculated for the general short form, with Cronbach Alpha = 0.85 and McDonald’s Omega = 0.90. This indicates that the 18-item scale offers high overall reliability. In terms of subdimensions, the IC, TA, and CC dimensions offer “good” reliability, while the AC, AI and EC dimensions fall within the “acceptable” range.

### 3.5 Data analysis

After the data obtained from the participants were checked, they were transferred to the RStudio statistical package. Among the personal variables included in the analyses, *gender* was coded as a categorical variable (female = 1, male = 2), *grade level* as a continuous variable (1–4), and *academic achievement* as a self-reported achievement level on a four-point scale (1 = poor, 4 = very good), which was treated as a continuous variable in the analyses. In the first stage, the normality of the data was examined; skewness and kurtosis values were found to be within the range of −1.5 to +1.5, indicating that the data met the assumption of normal distribution (Tabachnick and Fidell, 2013). Descriptive statistics for the subscales were calculated to define the academic procrastination levels of pre-service teachers, and given that the subscale scores were continuous and met the normality assumption, Pearson correlation analysis was conducted to examine the relationship patterns between the subscales.

A Structural Equation Model (*SEM*) was applied to examine the effects of personal variables on academic procrastination and the mediating role of academic confidence. All *SEM* analyses were conducted in the RStudio (2024.12.0) environment; the lavaan package was used in the modeling process. To assess the models’ fit with the data, fit indices such as *χ^2^/df, CFI, TLI, RMSEA*, and *SRMR* were reported. Model fit was evaluated using commonly recommended criteria: χ^2^/df ≤ 3, CFI and TLI values of 0.90 or above, and RMSEA and SRMR values of 0.08 or below were considered indicative of acceptable fit, whereas CFI and TLI values of 0.95 or above and RMSEA values of 0.06 or below were considered indicative of good fit (Hu and Bentler, 1999; Kline, 2016). Two structural models were estimated: a direct effects model (model 1) and a mediation model (model 2). Although preliminary analyses indicated that the data met the assumption of univariate normality, the MLR (robust maximum likelihood) estimator was used in Model 1 to obtain standard errors and fit statistics that remain robust to potential departures from multivariate normality. In Model 2, the ML estimator was adopted to enable bias-corrected bootstrap confidence intervals for the indirect effects, as bootstrap-based inference requires the ML estimator in the lavaan package. Direct path coefficients were reported using standardized beta (*β*) values.

Bootstrap-based mediation analyses with 5,000 resamples were conducted to test the mediating role of AC, with the significance of indirect effects assessed using 95% bias-corrected bootstrap confidence intervals; an effect was considered significant when the confidence interval did not include zero (Preacher and Hayes, 2008). Finally, Latent Profile Analysis (*LPA*) was applied to identify latent profiles of pre-service teachers based on their reasons for academic procrastination. Models containing two to five profiles were compared using the *tidyLPA* package (Rosenberg et al., 2018) under the equal variances specification (Model 1 in tidyLPA), which was preferred for its parsimony and estimation stability given the number of indicators and profiles examined. Model selection was guided by *AIC*, *BIC*, sample-size adjusted *BIC* (*SABIC*), entropy, and the Bootstrap Likelihood Ratio Test (*BLRT*). The most appropriate solution was determined based on the lowest information criteria, acceptable classification accuracy (Entropy ≥ 0.70), and significant *BLRT* improvement (*p* < 0.05) over the preceding model (Nylund et al., 2007). Model fit indices for all solutions are presented in Table 2.

**Table 2:** Latent profile analysis – model comparison (2–5 profiles).

Profiles	*LogLik*	*AIC*	*BIC*	*SABIC*	*Entropy*	*n_min*	*n_max*	*BLRT*	*p*
2	−3,949	7,935	8,188	7,955	0.731	0.331	0.669	443.3	0.0099
3	−3,897	7,847	8,194	7,874	0.681	0.149	0.516	102.3	0.0099
4*	−3,871	7,808	8,248	7,946	0.719	0.149	0.437	52.9	0.0099
5	−3,859	7,798	8,332	7,966	0.687	0.104	0.282	23.9	0.0297

* = Selected model (4 profiles). LogLik, log-likelihood; AIC, Akaike Information Criterion; BIC, Bayesian Information Criterion; SABIC, Sample-size Adjusted BIC; n_min/n_max, smallest/largest profile proportions; BLRT, Bootstrap Likelihood Ratio Test. Entropy ≥ 0.70 indicates acceptable classification accuracy (Nylund et al., 2007). BLRT *p* < 0.05 indicates significant improvement over k-1 profiles. *N* = 490.

All analyses were conducted with the data obtained from the 490 pre-service teachers, and the sample was not split for cross-validation purposes. This decision was based on considerations of statistical power and estimation stability. In SEM, larger samples provide more stable parameter estimates and more reliable fit statistics (Kline, 2016). Similarly, the accuracy of profile enumeration in LPA, including the performance of the bootstrap likelihood ratio test and the detection of relatively small latent profiles, improves as sample size increases (Nylund et al., 2007; Spurk et al., 2020). Splitting the sample would have approximately halved the number of cases available for each procedure and could have compromised the stability of both the measurement model and the profile solutions.

## 4 Results

### 4.1 Descriptive statistics and correlations for academic procrastination reasons

In the results section, descriptive statistical findings regarding the academic procrastination tendencies of pre-service teachers are presented first. In accordance with the first research question of the study, the mean, standard deviation, and correlation values for each dimension were calculated (Table 3).

**Table 3:** Descriptive statistics and intercorrelations among study variables.

Subscale	*N*	*M*	*SD*	1	2	3	4	5	6
TA	490	3.28	1.00	—					
EC	490	2.52	1.06	0.36***	—				
IC	490	3.07	1.04	0.38***	0.43***	—			
CC	490	3.21	1.05	0.50***	0.32***	0.32***	—		
AC	490	3.13	0.92	0.30***	0.22***	0.26***	0.30***	—	
AI	490	2.95	0.94	0.37***	0.33***	0.36***	0.35***	0.35***	—

*p < 0.05, **p < 0.01, ***p < 0.001.

According to the data presented in Table 3, the highest average scores are obtained in the TA (*M* = 3.28, *SD* = 1.00) and CC (*M* = 3.21, *SD* = 1.05) dimensions, with AC (*M* = 3.13, *SD* = 0.92) closely follows these. The lowest average score was obtained in the EC dimension (*M* = 2.52, *SD* = 1.06). According to the correlation analysis findings, significant positive relationships were identified among all subscales. While the strongest correlation was observed between TA and CC (*r* = 0.50, *p* < 0.001), the AC dimension exhibited relatively lower correlations with other subscales, ranging between *r* = 0.22 and 0.35.

### 4.2 Structural equation modeling: direct and mediating effects

The structural equation model (*SEM*) was conducted to test the hypothesized direct and indirect effects of personal variables on academic procrastination dimensions through the mediating role of AC.

#### 4.2.1 Model fit indices

Various fit indices were calculated to evaluate the fit of the proposed *SEM* to the data and are presented in Table 4.

**Table 4:** Model fit indices for the structural equation model.

Fit index	Value	Recommended threshold	Interpretation
CFI	0.925	≥0.90	Acceptable
TLI	0.910	≥0.90	Acceptable
RMSEA	0.048	≤0.06	Good Fit
SRMR	0.044	≤0.08	Good Fit
*χ*^2^ / df	2.450	≤3.00	Acceptable

CFI, Comparative Fit Index; TLI, Tucker–Lewis Index; RMSEA, Root mean square error of approximation; SRMR, Standardized root mean square residual.

**Figure 1:**
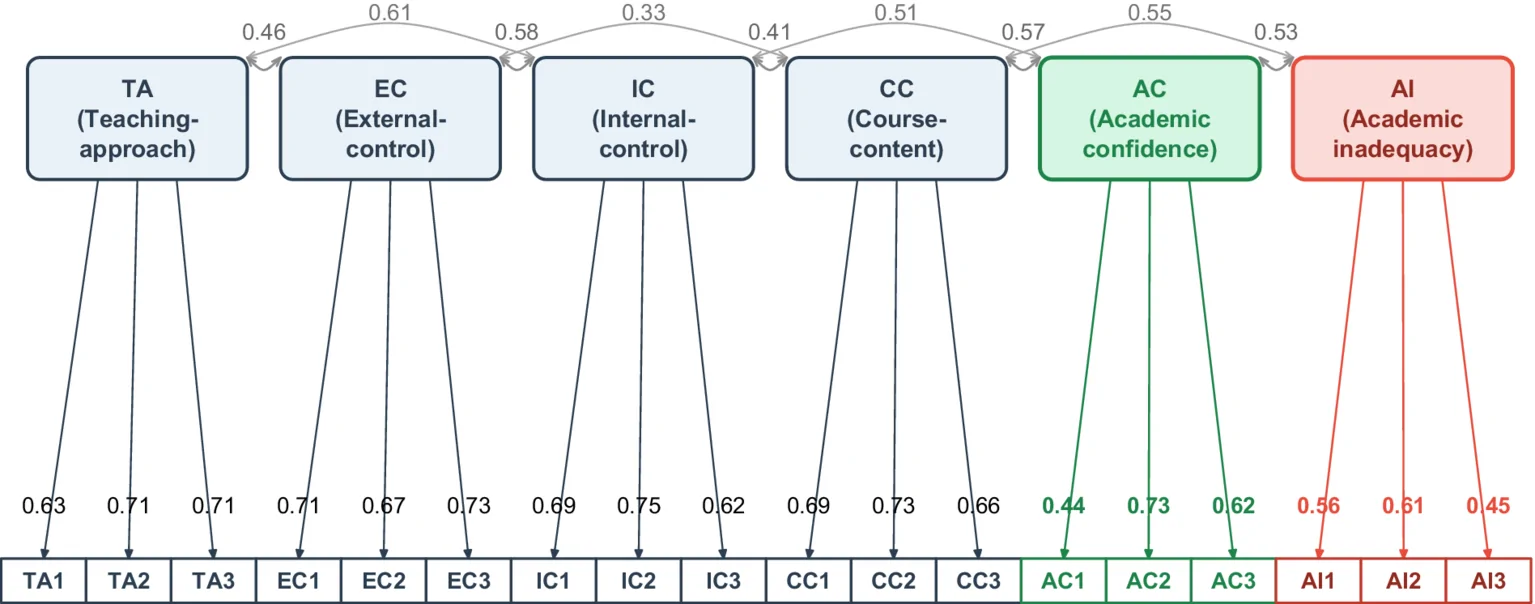
CFA measurement model - standardized factor loadings. TA, Teaching-approach-based reasons; EC, External-control-based reasons; IC, Internal-control-based reasons; CC, Course-content-based reasons; AC, Academic-confidence-based reasons; AI, Academic-inadequacy-based reasons. All paths shown are statistically significant (*p* < 0.05). Standardized coefficients (*β*) are shown.

According to Table 4, model fit statistics indicate that the model demonstrated an acceptable to good fit to the data (*CFI* = 0.925; TLI = 0.910; *RMSEA* = 0.048; *SRMR* = 0.044). Furthermore, the χ^2^/df ratio of 2.45 indicates that the model is also acceptable in terms of parsimony. The factor structure and standardized loadings of the measurement model are presented in Figure 1.

Figure 1 presents the *CFA* measurement model with six latent constructs (TA, EC, IC, CC, AC, AI) and their respective observed indicators (e.g., TA1-TA2-TA3), with standardized path coefficients. Factor loadings ranged from 0.44 to 0.75, indicating that all indicators adequately represented their respective latent constructs.

#### 4.2.2 Structural relationships and path coefficients

In the study, the relationships between personal variables (gender, grade level, and academic achievement) and the sub-dimensions of academic procrastination were first examined within the *SEM* framework, in line with H1. The direct standardized path coefficients for the model are presented in Table 5.

**Table 5:** Direct path coefficients in the structural equation model.

Path	*B*	*SE*	*z*	*p*	*β*
Gender → TA	−0.299	0.096	−3.124	0.002	−0.172**
Gender → EC	0.071	0.112	0.633	0.527	0.034
Gender → IC	−0.142	0.117	−1.219	0.223	−0.074
Gender → CC	−0.035	0.108	−0.326	0.745	−0.017
Gender → AC	0.037	0.068	0.550	0.583	0.033
Gender → AI	−0.312	0.099	−3.157	0.002	−0.200**
Grade → TA	0.072	0.042	1.723	0.085	0.096
Grade → EC	0.108	0.052	2.093	0.036	0.120*
Grade → IC	0.040	0.047	0.868	0.385	0.049
Grade → CC	0.205	0.052	3.920	0.000	0.236***
Grade → AC	0.024	0.030	0.823	0.410	0.049
Grade → AI	0.049	0.042	1.188	0.235	0.073
Achievement → TA	−0.030	0.053	−0.557	0.577	−0.032
Achievement → EC	−0.076	0.064	−1.185	0.236	−0.068
Achievement → IC	−0.168	0.062	−2.709	0.007	−0.163**
Achievement → CC	−0.003	0.062	−0.044	0.965	−0.002
Achievement → AC	0.044	0.041	1.088	0.276	0.072
Achievement → AI	−0.062	0.054	−1.153	0.249	−0.073

*N* = 490. Estimates were obtained using the MLR (robust maximum likelihood) estimator. *p < 0.05, **p < 0.01, ***p < 0.001.

**Figure 2:**
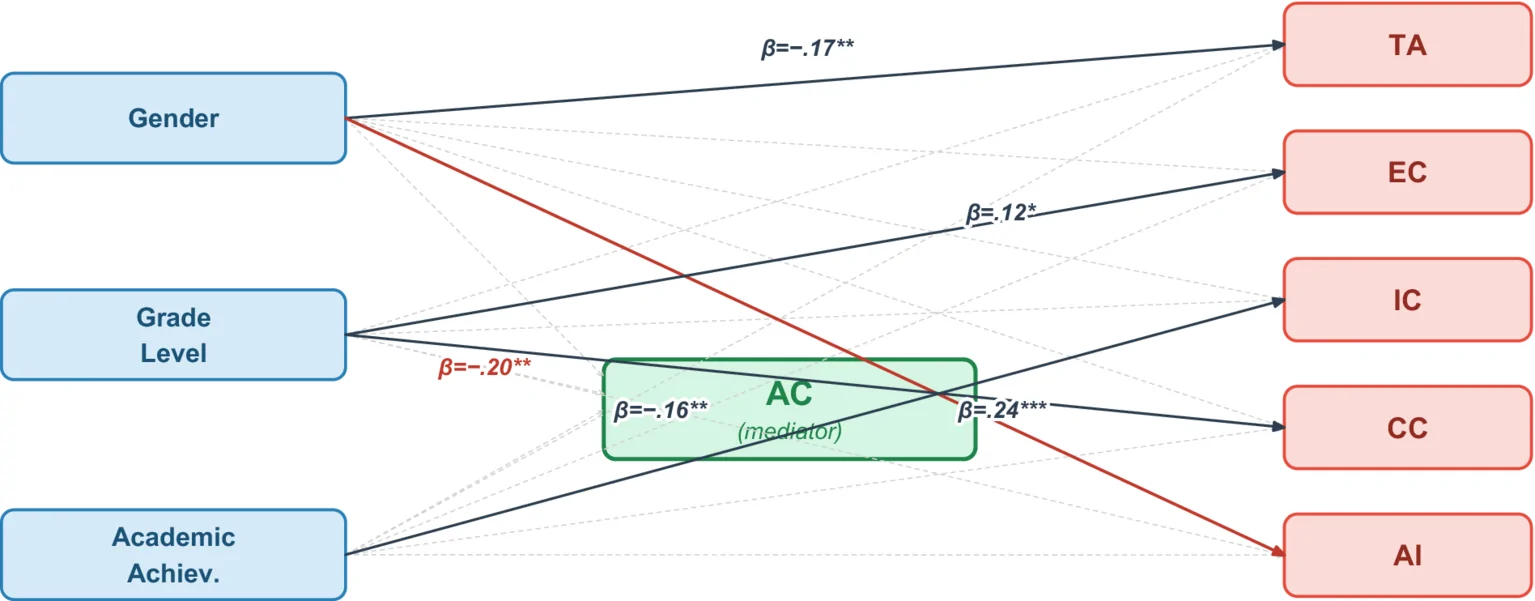
Standardized structural equation model – direct effects (Model 1). Solid lines = significant paths (*p* < 0.05) Dashed = non-significant AC, academic-confidence-based reasons (mediator) *β* values shown *p < 0.05, **p < 0.01, ***p < 0.001.

According to Table 5 (Model 1); gender (female = 1, male = 2) negatively predicted teaching-approach-based reasons (TA; *β* = −0.17, *p* = 0.002) and academic-inadequacy-based reasons (AI; *β* = −0.20, *p* = 0.002). While grade level positively predicted external-control-based reasons (EC; *β* = 0.12, *p* = 0.036) and course-content-based reasons (CC; *β* = 0.24, *p* < 0.001), academic achievement negatively predicted internal-control-based reasons (IC; *β* = −0.16, *p* = 0.007). None of the personal variables significantly predicted academic-confidence-based reasons (AC). These findings indicate that H1 was partially supported. The overall pattern of direct path coefficients is presented in Figure 2.

These findings regarding direct effects necessitate a more detailed examination of AC’s mediating role within the *SEM* framework. Accordingly, Table 6 presents the bootstrap-based results (5,000 resamples) for the mediating role of AC in the relationship between personal variables and the subdimensions of academic procrastination.

**Table 6:** Structural equation modeling results for the mediation model (bootstrap = 5,000).

Outcome	Predictor	*B*	*SE*	*z*	*p*	*CI_low*	*CI_up*	*β*
AC	Gender	0.037	0.071	0.527	0.598	−0.107	0.172	0.033
AC	Grade	0.024	0.031	0.796	0.426	−0.034	0.091	0.049
AC	Achievement	0.044	0.041	1.076	0.282	−0.034	0.130	0.072
TA	AC	0.627	0.139	4.509	<0.001	0.406	0.952	0.414***
TA	Gender	−0.322	0.095	−3.373	0.001	−0.520	−0.147	−0.186**
TA	Grade	0.056	0.042	1.334	0.182	−0.027	0.139	0.075
TA	Achievement	−0.057	0.053	−1.085	0.278	−0.165	0.043	−0.061
EC	AC	0.605	0.159	3.803	<0.001	0.345	0.972	0.333***
EC	Gender	0.048	0.110	0.437	0.662	−0.170	0.260	0.023
EC	Grade	0.093	0.053	1.757	0.079	−0.012	0.193	0.104
EC	Achievement	−0.103	0.062	−1.655	0.098	−0.232	0.014	−0.092
IC	AC	0.652	0.147	4.439	<0.001	0.423	1.008	0.390***
IC	Gender	−0.167	0.112	−1.488	0.137	−0.410	0.033	−0.087
IC	Grade	0.025	0.047	0.524	0.601	−0.062	0.119	0.030
IC	Achievement	−0.197	0.062	−3.196	0.001	−0.326	−0.083	−0.190**
CC	AC	0.745	0.182	4.082	<0.001	0.455	1.180	0.424***
CC	Gender	−0.063	0.105	−0.597	0.550	−0.272	0.143	−0.031
CC	Grade	0.187	0.053	3.531	<0.001	0.086	0.292	0.215***
CC	Achievement	−0.036	0.064	−0.562	0.574	−0.160	0.088	−0.033
AI	AC	0.733	0.140	5.244	<0.001	0.481	1.030	0.537***
AI	Gender	−0.340	0.096	−3.530	<0.001	−0.535	−0.157	−0.218***
AI	Grade	0.032	0.043	0.735	0.462	−0.057	0.112	0.047
AI	Achievement	−0.094	0.053	−1.771	0.077	−0.198	0.014	−0.112

*N* = 490. B, unstandardized coefficient; β, standardized coefficient; CI, 95% bias-corrected bootstrap confidence interval (5,000 resamples). ML estimator was used. *p < 0.05, **p < 0.01, ***p < 0.001.

**Figure 3:**
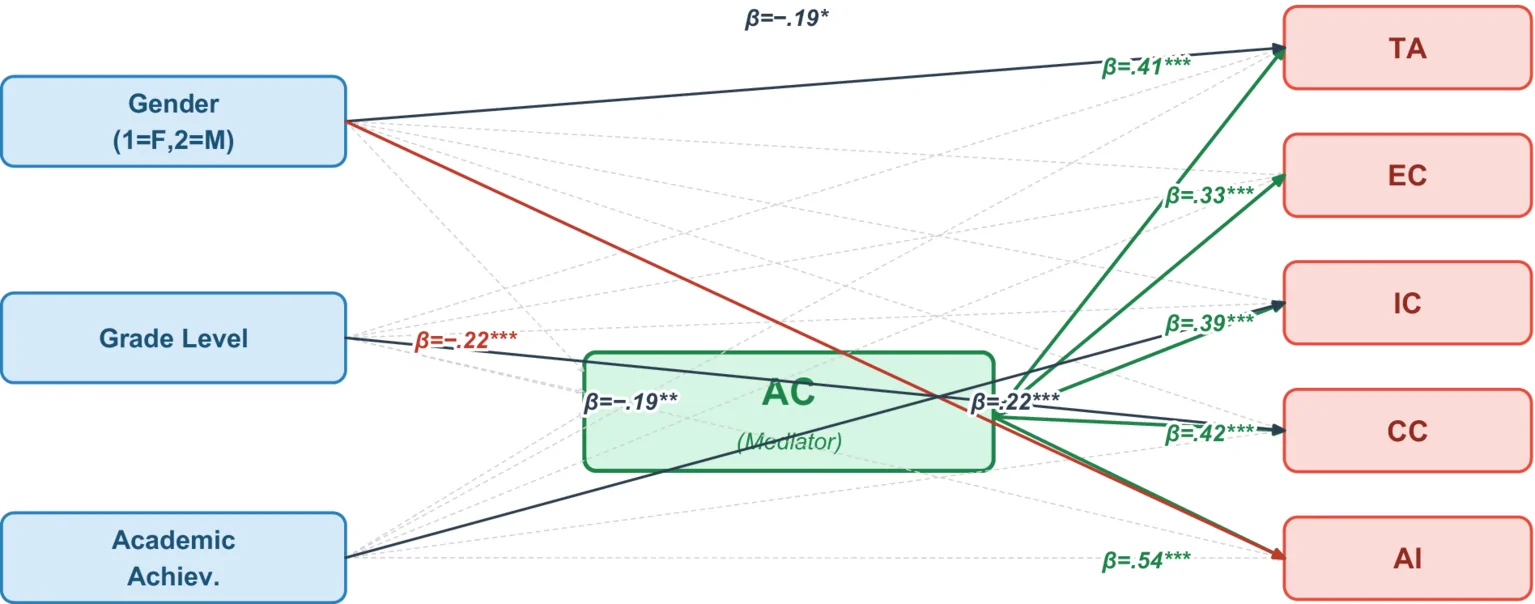
Standardized SEM - mediation model (Model 2). Green arrows represent b-paths from AC to procrastination dimensions. Dashed lines indicate non-significant a-paths (personal variables → AC). Standardized coefficients (β) are shown. Bootstrap = 5,000, ML estimator. *p < 0.05, **p < 0.01, ***p < 0.001.

Table 6 presents the results of Model 2, in which academic-confidence-based reasons (AC) were tested as a mediator between personal variables and procrastination dimensions (Bootstrap = 5,000, *ML* estimator). The paths from personal variables to AC (a-paths) were all non-significant (gender: *β* = 0.03, *p* = 0.598; grade: *β* = 0.05, *p* = 0.426; achievement: *β* = 0.07, *p* = 0.282), indicating that the conditions for mediation were not met and H2 was not supported. Nevertheless, AC significantly and positively predicted all five procrastination dimensions (b-paths): TA (*β* = 0.41, *p* < 0.001), EC (*β* = 0.33, *p* < 0.001), IC (*β* = 0.39, *p* < 0.001), CC (*β* = 0.42, *p* < 0.001), and AI (*β* = 0.54, *p* < 0.001). Figure 3 visually demonstrates that the paths from personal variables to AC were non-significant, whereas AC strongly predicted all procrastination dimensions.

### 4.3 Determining different academic procrastination profiles

LPA was applied to determine latent profiles of pre-service teachers based on six academic procrastination dimensions. Models with two to five profiles were compared using *AIC, BIC, SABIC, entropy*, and *BLRT* (Table 2). The four-profile solution was selected based on the lowest SABIC (7946), adequate entropy (0.719), and significant *BLRT* improvement over the three-profile solution (*BLRT* = 52.9, *p* = 0.0099). Although the five-profile model yielded a marginally lower *AIC,* the *BLRT* was only marginally significant (*p* = 0.030) and entropy declined (0.687), indicating insufficient classification improvement. Therefore, the four-profile solution was retained as the most parsimonious and interpretable model.

Table 7, in turn, presents the mean scores for each procrastination dimension across the four identified profiles.

**Table 7:** Average scores on six latent dimensions by profile.

Profile	Label	*N*	*%*	TA	EC	IC	CC	AC	AI
1	Selective procrastinators	73	15	3.69	1.33	2.97	3.80	3.59	3.12
2	Moderate procrastinators	214	44	3.26	2.88	3.14	3.20	2.99	3.03
3	High-engaged procrastinators	96	20	4.18	3.81	4.01	4.04	3.82	3.59
4	Minimal procrastinators	107	22	2.23	1.48	2.14	2.07	2.49	2.10

According to the findings presented in Table 7, four distinct latent profiles have been identified regarding pre-service teachers’ reasons for academic procrastination. The profiles differ in terms of their mean scores on the sub-dimensions of reasons for academic procrastination.

The first profile (*Selective Procrastinators*) has relatively high averages in the TA (*M* = 3.69) and CC (*M* = 3.80) dimensions, while exhibiting the lowest average in the EC dimension (*M* = 1.33).

The second profile (*Moderate Procrastinators*) is characterized by moderate average scores across all subdimensions of academic procrastination reasons.

The third profile (*High-Engaged Procrastinators*) has the highest average scores in all sub-dimensions, particularly in TA (*M* = 4.18), CC (*M* = 4.04), IC (*M* = 4.01), and AC (*M* = 3.82).

The fourth profile (*Minimal Procrastinators*) shows the lowest average scores in all subdimensions of academic procrastination reasons (CC: *M* = 2.07; AC: *M* = 2.49).

When examining the sub-dimensions, significant average differences between profiles were particularly evident in the AC, AI, TA, and CC dimensions; the IC and EC dimensions also showed variability between the profiles.

Table 2 presents the model fit indices for the two- to five-profile solutions.

**Figure 4:**
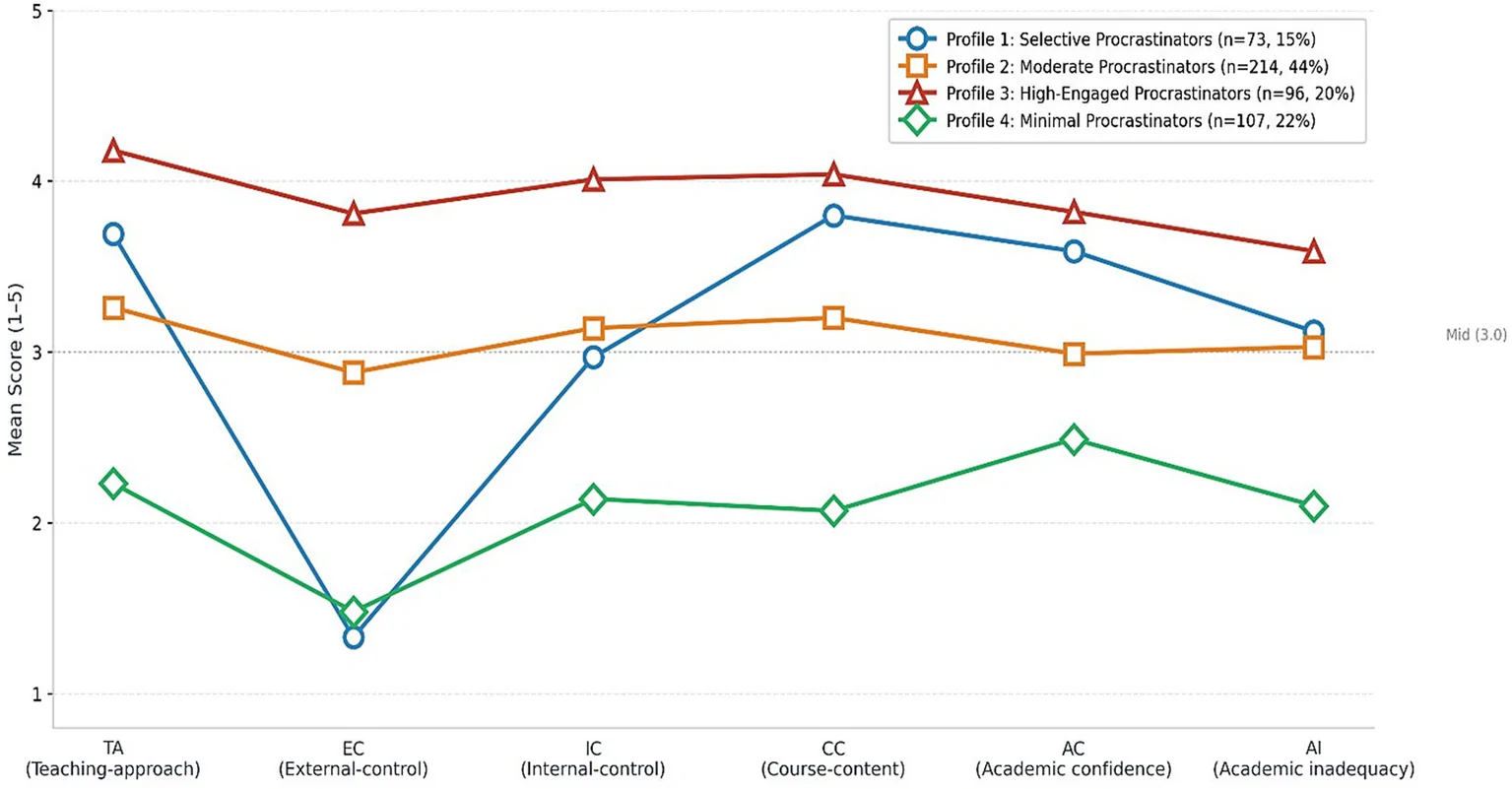
Average scores for academic procrastination dimensions across four profiles.

To visually examine how the latent profiles of pre-service teachers, determined according to academic procrastination reasons, differ in terms of average scores on subdimensions, profile-based average distributions are presented in Figure 4.

As can be seen from Figure 4, the differentiation between profiles is particularly evident in the TA, CC and AC dimensions. The findings reveal that pre-service teachers’ reasons for academic procrastination exhibit a multidimensional and heterogeneous structure.

## 5 Discussion

Because a cross-sectional design was employed in this study, all relationships reported below are interpreted as associations (correlations) between variables rather than as cause-and-effect or temporal effects. The descriptive findings examining the distribution of and correlations among reasons for academic procrastination, which constituted the first research question, revealed that pre-service teachers showed a moderate tendency to procrastinate across most dimensions, with these reasons exhibiting positive and significant interrelationships. The relatively higher procrastination tendencies observed in the TA and CC dimensions suggest that procrastination is more closely associated with pedagogical and contextual components. This finding is consistent with previous research demonstrating that procrastination levels decrease when educators hold high expectations, provide students with autonomy, and when students strive to understand course material (Corkin et al., 2014; Freeman et al., 2007; Khalifa, 2023; Ryan and Deci, 2020). Notably, the strong correlation between TA and CC (*r* = 0.50) indicates that these dimensions may exert overlapping and mutually reinforcing in their associations with procrastination behavior. The comparatively lower correlations observed for AC are particularly noteworthy; this pattern suggests that academic-confidence-based procrastination is qualitatively distinct from other avoidance-based reasons and represents an independent psychological construct consistent with active procrastination theory (Choi and Moran, 2009; Habelrih and Hicks, 2015). Collectively, these findings further demonstrate that academic procrastination reflects interrelated yet distinct underlying reasons rather than a homogeneous psychological structure (Liu et al., 2025; Sparfeldt and Schwabe, 2024).

The *SEM* results testing the first hypothesis revealed a selective rather than a pervasive pattern, where personal variables predicted specific procrastination dimensions, thereby providing partial support for H1. These findings suggest that procrastination among pre-service teachers is associated with different reasons depending on their personal characteristics. Gender was a significant predictor for TA and AI; grade level for EC and CC; and academic achievement for IC. Specifically, gender played a distinctive role in sensitivity to teaching approaches and self-perceptions of academic inadequacy, with female pre-service teachers reporting higher tendencies in these areas. Regarding grade level, it was found to be a differentiating factor for procrastination reasons linked to limited family and teacher support mechanisms and the perceived disconnect between course content and professional context. In terms of academic achievement, pre-service teachers with higher success levels reported lower IC-based procrastination, involving time management, study habits, and academic social circles. This finding is consistent with Bahl et al. (2025), Rakes et al. (2013), and Wang et al. (2025), supporting the view that academic achievement serves as an indicator of self-regulation capacity (Choy and Cheung, 2018). Notably, the finding that no personal variable was significantly associated with AC aligns with the correlation findings showing AC’s divergence from other dimensions. This suggests that AC-based procrastination may be related to psychological constructs independent of the examined demographics, providing a basis for future research.

Regarding the second hypothesis (H2), the mediation analyses conducted via the *SEM* framework revealed that academic confidence (AC) did not serve as a significant mediator between personal variables and academic procrastination dimensions. This result reflects the finding that personal variables were not significantly associated with AC. Although AC was a powerful and direct predictor of all procrastination sub-dimensions, it was independently associated with these dimensions rather than serving as a pathway linking demographic variables to them. This suggests that the associations of gender, grade level, and academic achievement with procrastination reasons are not accounted for by academic confidence. Instead, AC appears to be a stable construct that is relatively independent of the demographic variables examined in this study, functioning as a central correlate of procrastination behavior in its own right. This situation is consistent with the mixed procrastination profiles identified in the *LPA* findings. The emerging pattern is consistent with a Dunning–Kruger–type dynamic (Kruger and Dunning, 1999), in which overestimation of one’s capacity may coexist with perceptions of inadequacy. Because the data are cross-sectional, this interpretation is offered as a theoretical possibility rather than an observed temporal sequence. This pattern aligns with miscalibrated confidence (Bol and Hacker, 2001; Maki et al., 2005) and failures in the “forethought” phase of Zimmerman’s (2000) self-regulation theory. Among students who underestimate or cannot realistically analyze a task, this self-regulatory pattern was most strongly associated with perceptions of inadequacy (AI) and was also associated with the endorsement of external/internal control (EC, IC) and the instructional context (TA, CC) as a rationalization. This pattern, given the cross-sectional nature of the data, is best understood as reflecting co-occurring tendencies rather than a sequential process. These results suggest that procrastination may be a dynamic behavior reconstructed within shifting cognitive appraisals rather than a static trait; however, this interpretation should be evaluated considering the limitations of cross-sectional data.

In line with the second research question, findings regarding procrastination reason-based profiles identified four distinct groups among pre-service teachers.

*Profile-1 (Selective Procrastinators, 15%)* is characterized by high levels of AC; these individuals primarily justify their procrastination through TA and CC while rejecting the externalization of control (EC). Despite acknowledging certain inadequacies, they believe they can manage the process but engage in “selective” procrastination when they find the content or methodology unsatisfactory. This finding largely aligns with the “discontent with studies” type identified by Grunschel et al. (2013), where individuals attribute procrastination to situational and contextual factors such as instructional design and educator support rather than personal shortcomings. Similarly, Gross et al. (2024) demonstrated that the “externalizing” type exhibits characteristics largely overlapping with selective procrastinators, also showing a trend toward lower academic achievement.

*Profile-2 (Moderate Procrastinators, 44%)* constitutes the majority of the sample and reflects the prevalent and normative landscape of academic procrastination among pre-service teachers, as evidenced by its average scores across all dimensions. This finding is largely consistent with the literature; previous profile studies on diverse samples also report moderate procrastinators as the most frequent profile (Gross et al., 2024; Rist et al., 2023; Rozental et al., 2015; Zhou et al., 2024; Xu, 2022). This profile manages procrastination behavior with moderate confidence and balanced justifications, exhibiting a structure that is neither as bold as active procrastinators nor as detached as the low-engagement group. Supporting this, Rozental et al. (2015) noted that the “Average Procrastinators” group one of five profiles in their study scored at moderate levels across psychological indicators such as anxiety, depression, and quality of life. In contrast, Ozberk and Türk-Kurtça (2021) identified moderate procrastinators among Turkish university students as a group experiencing indifference toward academic tasks and a lack of belonging and motivation, whereas no such moderate profile was found among Balkan students. These findings, which highlight cultural differences in procrastination, do not align with the results of the current study.

*Profile-3 (High-Engaged Procrastinators, 20%)* displays the highest scores in both AC and all other procrastination dimensions, bearing conceptual similarities to the active procrastinator profile in the literature. The simultaneous display of high AC and high scores across all procrastination dimensions suggests that this group utilizes all available rationalizations to justify their procrastination behavior. This pattern is consistent with the *SEM* findings, in which AC was strongly associated with all procrastination dimensions. Similar profiles in the literature overlap with the “successful pressure-seeking” group (Grunschel et al., 2013), who use time pressure as a source of motivation, and “unconcerned delayers” (Rist et al., 2023), who achieve their goals despite procrastination. While Rozental et al. (2015) emphasize that such profiles may be associated with low depression and high quality of life, Gross et al. (2024) point out that high levels of procrastination justification may increase the risk of burnout and imposter syndrome in the long term. Given the cross-sectional design, however, this resemblance to active procrastination can be interpreted not as evidence that these students deliberately and strategically delay their work, but as a descriptive parallel.

*Profile-4 (Minimal Procrastinators, 22%)* possesses the lowest scores across all procrastination dimensions, representing the segment that tends to complete academic tasks on time. Low AC scores indicate that this group does not exhibit confidence-based procrastination, while low AI scores suggest that procrastination rooted in perceptions of inadequacy is not prominent in this group. Furthermore, low scores regarding pedagogical factors and control mechanisms reveal that this group largely does not adopt the typical procrastination justifications defined in the scale. These findings show similarities with profiles identified in the literature as “inconspicuous type” (Grunschel et al., 2013), “high regulated” (Rebetez et al., 2015), “occasional delayers/fast performers” (Rist et al., 2023), and “efficient learners” (Xu, 2022). In the literature, these profiles are characterized by course satisfaction, success in emotion and time management, and high levels of self-regulation and self-esteem. In this respect, Profile 4 empirically illuminates the “timely-performing” student segment, which is less popular in procrastination literature and can be considered the normative opposite of procrastination.

## 6 Theoretical implications

This study examined the academic procrastination behaviors of pre-service teachers by integrating variable-centered (SEM) and person-centered (LPA) approaches. Through this holistic framework, the multidimensional nature of procrastination reasons, the role of personal variables, the predictive function of academic confidence (AC), and heterogeneous procrastination profiles were analyzed. Procrastination reasons were addressed within a psycho-pedagogical context across six dimensions: TA, CC, EC, IC, AC, and AI. The study offers a novel contribution to the literature by examining academic procrastination behavior through the joint consideration of “active procrastinators,” characterized by a preference for risk-taking and working under time pressure within the context of AC, and “passive procrastinators,” characterized by low self-confidence stemming from perceived AI. The *SEM* results revealed that the relationships between personal variables and procrastination reasons are multidimensional, highlighting AC-based procrastination as a central component strongly associated with the other justifications. Furthermore, the findings indicated a pattern conceptually aligned with the Dunning–Kruger effect. These results demonstrate consistency with *Active Procrastination Theory* (Chu and Choi, 2005), *Self-Regulated Learning Theory* (Zimmerman, 2000), and *Self-Determination Theory* (Ryan and Deci, 2000).

## 7 Conclusion and implications

The findings of this study were derived from an integrated analytical process extending from descriptive and correlational analyses to *SEM* and ultimately to *LPA*, with the results at each stage consistently supporting one another. When these analyses are evaluated collectively, it becomes evident that academic procrastination among pre-service teachers is not a uniform psychological tendency. Instead, it is understood to be a heterogeneous and dynamic behavioral system shaped by diverse motivational foundations, personal characteristics, and cognitive patterns.

Descriptive findings indicated a moderate prevalence across all procrastination dimensions, while relatively higher means in TA and CC revealed that procrastination is more closely linked to pedagogical and contextual factors. Correlational analyses showed that AC exhibited the lowest relationship with other reasons. This represents more than a statistical divergence; it indicates that AC-based procrastination is a qualitatively distinct psychological construct from other avoidance-based reasons. This pattern also manifests across the four profiles identified in the *LPA*: *Profile-3*, with the highest AC, also had the highest scores in all other dimensions, whereas *Profile-4*, with the lowest AC, showed the lowest procrastination tendency across the board. This cross-pattern consistently underscores the central position of AC within the procrastination system across both variable- and person-centered analyses.

SEM findings further deepen this picture. The pattern of direct associations between personal variables and procrastination dimensions appears to be selective. Specifically, female pre-service teachers reported higher procrastination in TA and AI; EC and CC based procrastination was higher among students at higher grade levels, whereas IC-based procrastination was lower among those with higher academic achievement. This selective pattern gains significance when cross-referenced with *LPA* profiles: the increase in CC and EC-based procrastination with grade level aligns with *Profile-3*’s tendency to utilize all justifications simultaneously; likewise, the decrease in IC-based procrastination with academic achievement overlaps with *Profile-4*’s self-regulatory structure. Conversely, AC was not significantly associated with any personal variable. This suggests that the examined demographics are limited in explaining individual differences in AC-based procrastination, indicating that this reason may remain consistent across different demographic groups.

Mediation analysis provides the most strategic insight: AC did not mediate the relationship between personal variables and procrastination dimensions; instead, it was powerfully and independently associated with all dimensions in the model. The fact that AC showed its strongest association with AI suggests that high academic confidence and justifications based on perceived inadequacy can coexist within the same individual. This dynamic is concretely observed in *Profile-1*, which justifies procrastination through TA and CC while also exhibiting relatively high AI scores. Unlike *Profile-1*, *Profile-2* presents a more balanced procrastination profile without a marked divergence between dimensions. *Profile-3* represents the most advanced form of this dynamic. By having the highest scores across all dimensions, this group’s simultaneous rationalization paired with high confidence presents a picture that conceptually overlaps with the Dunning–Kruger Effect.

These integrated findings suggest that strategies to address procrastination in teacher education should be profile-based and AC-focused. For *Profile-1*, instructional design enhancements aimed at improving the pedagogical context are recommended; for *Profile-2*, programs strengthening self-regulation skills; for *Profile-3*, time–pressure management strategies and metacognitive calibration interventions; and for *Profile-4*, protective monitoring and selective support mechanisms. By adopting these targeted approaches, educator support systems can be tailored to the specific psychological and contextual needs of each student group.

## 8 Limitations and future research

This study has several limitations. First, the use of a cross-sectional design requires that causal inferences between variables be approached with caution. Accordingly, the relationships presented in the findings reflect the patterns of association observed within the time frame examined. In addition, the sample structure, comprising seven different primary school teacher education programs, limits the representativeness of the findings for the broader pre-service teacher population. Data collection through online self-report measures carries risks such as social desirability bias and limited control over data collection conditions. A further limitation concerns the measurement strategy: the 18-item short form of the Academic Procrastination Reasons Scale was derived through exploratory and confirmatory factor analyses conducted on the same sample that was subsequently used for the SEM and LPA analyses. Because no independent sample was available for cross-validation, this procedure may have capitalized on sample-specific variance, and the possibility of sample-dependent bias in both the measurement model and the structural findings cannot be ruled out. Although the short form was grounded in the theoretical structure of the original 43-item scale and demonstrated acceptable fit and reliability, replication with a split-sample or holdout design, in which the instrument is refined on one subsample and validated on another, would provide a stronger safeguard against overfitting. Furthermore, due to the nature of the measurement instrument, the research focused on mental excuse-making mechanisms (cognitive and affective layers) rather than the operational frequency of procrastination. Profile identification was limited to the six dimensions of the instrument; thus, the profiles were defined within a relatively narrow theoretical framework as they did not incorporate personality traits, motivational factors, or other psychological processes emphasized in the procrastination literature. Future research is recommended to validate the short form in independent samples, examine procrastination profiles within a broader framework, investigate the underlying deep processes of procrastination rationalizations using qualitative and mixed methods, track transitions between profiles through longitudinal designs, and test the model across different cultural contexts.

## Data Availability

The raw data supporting the conclusions of this article will be made available by the authors, without undue reservation.
